# The Respiratory Mechanics of COVID-19 Acute Respiratory Distress Syndrome—Lessons Learned?

**DOI:** 10.3390/jcm13071833

**Published:** 2024-03-22

**Authors:** Rebecca L. Kummer, John J. Marini

**Affiliations:** 1 Department of Pulmonary and Critical Care Medicine, University of Minnesota School of Medicine, Minneapolis, MN 55455, USA; 2 Department of Pulmonary and Critical Care Medicine, Regions Hospital, St. Paul, MN 55101, USA

**Keywords:** COVID-19, ARDS, PEEP, VILI, PSILI

## Abstract

Acute respiratory distress syndrome (ARDS) is a well-defined clinical entity characterized by the acute onset of diffuse pulmonary injury and hypoxemia not explained by fluid overload. The COVID-19 pandemic brought about an unprecedented volume of patients with ARDS and challenged our understanding and clinical approach to treatment of this clinical syndrome. Unique to COVID-19 ARDS is the disruption and dysregulation of the pulmonary vascular compartment caused by the SARS-CoV-2 virus, which is a significant cause of hypoxemia in these patients. As a result, gas exchange does not necessarily correlate with respiratory system compliance and mechanics in COVID-19 ARDS as it does with other etiologies. The purpose of this review is to relate the mechanics of COVID-19 ARDS to its underlying pathophysiologic mechanisms and outline the lessons we have learned in the management of this clinic syndrome.

## 1. Introduction

Since its onset four years ago, the COVID-19 pandemic has challenged our way of doing things, both within and outside of the medical sphere. To date, there have been over 770 million confirmed cases of COVID-19 worldwide [1]. 

SARS-CoV-2, the virus responsible for COVID-19 disease, belongs to the *Coronavirus* family responsible for two other recent epidemics: Severe Acute Respiratory Syndrome (SARS) and Middle East Respiratory Syndrome (MERS). The coronaviruses responsible for COVID-19, SARS, and MERS are thought to have a natural reservoir in bats and infect humans through some intermediate host (Pangolins, Civet cats, and Dromedary camels, respectively) [2]. While the SARS and MERS epidemics involved about eight thousand and two thousand people, respectively, COVID-19 has proven much more transmissible, though thankfully less lethal with a mortality rate of approximately 2.3% compared to SARS (9.6%) and MERS (35.5%) [2]. Each virus is transmitted via respiratory droplets and/or close contact with an infected person. The clinical syndromes are highly similar, usually involving fevers, myalgias, and symptoms of pneumonia, which can progress to severe lung injury [2]. The viruses responsible for COVID-19 and SARS use the same host cellular receptor to gain access into host cells, ACE-2; however, the binding of SARS-CoV-2 to the ACE-2 receptor occurs with 10–20 times the binding capacity as compared to SARS-CoV, which may partially explain the increased transmissibility of SARS-CoV-2 [2]. In all three infections, the lungs are the primary target organ Indeed, the occurrence of three outbreaks due to coronaviruses within the past quarter century have taught us much about this genus of virus but at a steep cost. 

Of those patients with COVID-19 requiring hospitalization, approximately one third develop acute respiratory distress syndrome (ARDS) [3]. This unprecedented volume of patients with ARDS presented an opportunity for expanding our knowledge of the syndrome but required our approach to life support to adapt to unfamiliar respiratory challenges. Herein, we highlight the unique aspects of COVID-19 ARDS that have been observed to be relevant to lung mechanics and respiratory support, addressing associated clinical questions stemming from those observations that are highly relevant to effectively managing COVID-19 ARDS. We contend that the answers to these clinical questions often lie in careful observation and interpretation of the patient’s individual respiratory mechanics. 

## 2. Defining ARDS 

The Berlin criteria for the diagnosis of ARDS were developed via expert consensus in 2011 and include the onset of hypoxemia and bilateral opacities on chest imaging over the course of one week or less that cannot be fully explained by fluid overload [4]. More recently, a recommendation was made to expand the already-lenient Berlin criteria even further so that they might apply to a global population with different levels of access to certain resources such as invasive mechanical ventilation. The recommended expansions to the ARDS criteria are the inclusion of patients on high-flow nasal cannula (HFNC) at a flow rate of 30 L/min or more and allowing oxygen saturation to be measured via either pulse oximetry or blood gas analysis [5]. Given such broad definitions, multiple different pathologic mechanisms for the generation of hypoxemia may primarily initiate or strongly contribute to this clinical syndrome. Alveolar collapse or filling that leads to perfusion of poorly ventilated alveoli is the pathological hallmark of ARDS. Consequently, impairment in gas exchange usually correlates well with the amount of involved lung tissue, such that the responses of oxygenation and tissue compliance to medical interventions are generally proportional to one another [6]. However, mechanisms of hypoxemia other than lung unit collapse, edema, or consolidation do occur within the wide spectrum of ARDS, in some conditions more than others. 

## 3. Understanding Hypoxemia in COVID-19 ARDS

The histopathology of COVID-19 pulmonary disease aids in understanding the mechanisms of hypoxemia in this viral syndrome. SARS-CoV-2 is an enveloped, single-stranded RNA virion which encodes various structural, non-structural, and accessory proteins important for its life cycle [7]. Especially pertinent to this discussion, the spike-surface protein is responsible for fusing with target host cells. Angiotensin signaling is a biochemical pathway that appears to be central in COVID-19 pathogenesis. While ACE2 is present along the epithelium of the entire respiratory tract, it is highly abundant in the vascular endothelium and type 2 pneumocytes. Using the spike protein on its surface, the SARS-CoV-2 virus binds to ACE2, unlocking protective barriers to entry and the infection of cells. Hence, ACE2 acts as a cellular doorway—a receptor—for SARS-CoV-2 [8]. Binding to and entering host cells is a complicated process unique to each virus and host; while we know this crucial pathologic step involves several other key interactions such as S protein priming by serine protease TMPRSS2 [8] and host cell co-receptor interactions such as ACE-2 and Nrp1 [9], the intricacies of these interactions are beyond the scope of this review. The virus most likely enters the human host through the nasopharynx and trachea. In some potential patients, the innate immune system response to the infection stops the virus at this point and further dissemination is prevented. However, if the immune system response is inadequate, the virus may disseminate along the tracheobronchial tree to the alveolar level. There, it primarily infects type II pneumocytes. Viral replication leads to apoptosis of type II pneumocytes, decreased surfactant production, and the induction of inflammatory cascades [7]. Determining which factors determine who will go on to develop this exaggerated inflammatory response is beyond the scope of this review, but these likely have to do with the timing and strength of type I and type III interferon responses by the host [7]. 

Consistent with traditional (non-COVID) ARDS, the predominant histopathologic pattern seen upon post-mortem examination of the lungs of persons with COVID-19 pneumonia is diffuse alveolar damage (DAD) with alveolar edema and collapse, hyaline membrane formation, interstitial edema and capillary congestion, and loss of type II pneumocytes [10]. In one study of six lung-biopsied patients with COVID-19, the predominant histologic pattern was not DAD but rather acute fibrinous and organizing pneumonia (AFOP) without hyaline membrane formation; however, these patients represent a minority of cases and underwent needle biopsy sampling, which may have missed other histopathologies [11]. Nonetheless, even when DAD was the predominant histologic pattern, the majority of cases were noted to have areas of organizing fibrosis, suggesting early transition to the organizing phase of DAD [12]. Whether distinguished by AFOP or DAD with the presence of organizing pneumonia, the existence of fibroblast and loose collagen aggregates may explain the favorable response of COVID-19 ARDS to glucocorticoids. Distinct from other forms of ARDS, autopsied patients with COVID-19 ARDS have been noted to have an increase in pulmonary microthrombi [10,12]. Post-mortem examination of the vascular features of COVID-19 pneumonia using diverse techniques such as immunohistochemical staining, micro-computed tomographic imaging, electron microscopy, and corrosion casting demonstrated distinct vascular pathologies: diffuse endothelial damage due to endothelial cell viral invasion, thrombosis of alveolar capillaries, and intussusceptive angiogenesis [13]. Disrupted pulmonary vasoregulation may be a consequence of vascular involvement throughout the advancing course. Such processes may contribute to the extensive loss of aerated volume and the transition to very low tidal compliance in the advanced stages of unresolving disease. Given this unique pathophysiology with a focus on the vascular compartment, a discussion of how the mechanisms of hypoxemia may influence our clinical observations and the time course of COVID-19 pulmonary disease is warranted. This will serve as the basis for understanding certain puzzling mechanics of COVID-19 ARDS.

**Figure 1:**
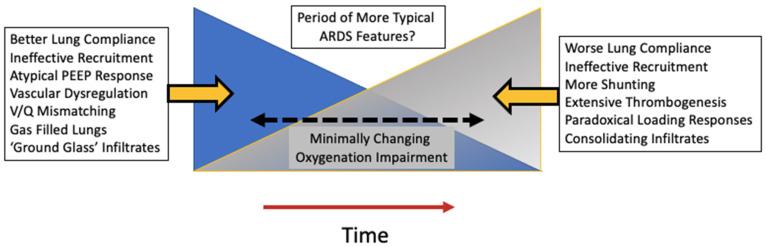
Changing time course of the underlying characteristics of COVID-19 ARDS. Early on in the disease course, lung compliance may be relatively well preserved, leading to atypical responses to recruitment maneuvers and PEEP titration. As time passes, the more typical features of ARDS physiology may develop including poor compliance and worsening shunting.

**Figure 2:**
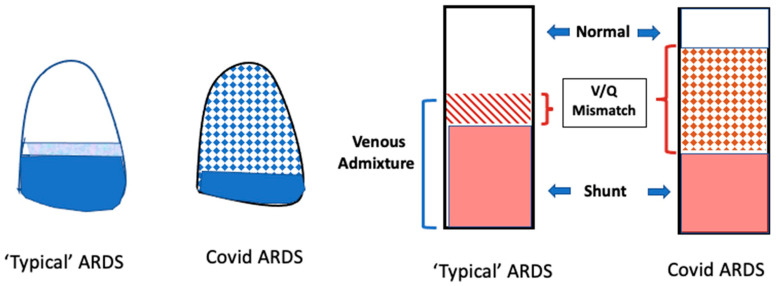
Mechanisms of gas exchange impairment in COVID-19 ARDS versus traditional ARDS. On the left, typical ARDS is depicted by a large area of dense consolidation (dark blue) representing shunt and a small area of less densely consolidated lung (light blue) representing V/Q mismatch. The white area represents unaffected lung (the baby lung). In COVID-19 ARDS, there is a relatively larger portion of V/Q mismatch distributed throughout much of the lung (checkered pattern). On the right side of the figure, a similar concept is demonstrated in a bar graph.

As with many other respiratory infections, symptoms and respiratory system mechanics in COVID-19 pneumonia evolve with time (Figure 1). Early in the course of infection, patients are more likely to have fewer infiltrates on CT scans, relatively unrestricted lung volumes, and near-normal compliance [14]. Despite a seemingly low burden of alveolar infiltrate and edema, these patients often present with profound hypoxemia. This interesting phenomenon was observed early in the COVID-19 pandemic experience and has been referred to as “happy” or “silent” hypoxemia. Chiumello and colleagues published a study of 32 patients with COVID-19 and compared them to typical, non-COVID-19 ARDS cohorts—one matched per their PaO_2_/FiO_2_ ratios and another matched based on respiratory system compliance [15]. They found that the COVID-19 ARDS patients had better compliance and larger end-expiratory gas volumes than their non-COVID counterparts matched for PaO_2_/FiO_2_ ratios. Importantly, in COVID-19 ARDS patients, their venous admixture did not correlate well with their fraction of non-aerated tissue, as is true with traditional ARDS, suggesting that the underlying mechanism during the initial phase of COVID-19 pneumonia was predominantly V/Q mismatching (rather than true shunt) (Figure 2). Considering the increased extent of micro-thromboses in COVID-19 pneumonia in its terminal phase, V/Q mismatch seems to fit well as the plausible mechanism of hypoxemia that is disproportional to mechanical impairment, at least early in the disease course. Some patients improve at this stage and recover from their viral pneumonia, while others progress to a more traditional ARDS phenotype with increased pulmonary edema, increased lung weight, compressive atelectasis, lower compliance, and reduced end-expiratory lung volumes [14]. What drives the transition from the high-compliance to the low-compliance phenotype remains unknown. Certainly, infection severity and the host immune response play crucial roles, but these may not fully explain the development of progressive lung injury. One proposed mechanism in those with vulnerable parenchyma who breathe vigorously is that of patient self-inflicted lung injury (P-SILI) [16], which we will now briefly discuss as it relates to COVID-19 pneumonia. 

## 4. Respiratory Mechanics and Lung Injury

Ventilator-induced lung injury (VILI) is experimentally well-demonstrated and is been widely accepted clinical entity in which acute lung injury develops due to mechanical ventilation itself. Whether it relates primarily to excessive tidal volumes, excessive driving pressure, ventilation frequency, or, more likely, the damaging energy with each tidal cycle that causes intolerable strain and lung injury is actively studied and still debated [17]. The contributory roles of the vascular pressures and altered hemodynamics have been strongly suggested by controlled experiments [18]. Whatever the contributing factors; however, there is a general consensus from the critical care community that, when not titrated appropriately, the mechanical breaths delivered by the ventilator can harm vulnerable lung tissues [19]. A natural inquiry that may follow is whether harmful volumetric distortions or damaging energy also occur during spontaneous ventilation in a pre-injured lung. The development of lung injury following hyperventilation has been reported in large animal models [20]. In spontaneously breathing patients with increased respiratory drive, large tidal volumes applied to previously injured lungs with less aeratable capacity (the ‘baby lung’ of ARDS) may lead to damaging levels of local strain and exacerbate or perpetuate the lung injury [21]. Indeed, in selected patients who underwent a trial of non-invasive ventilation (NIV) for acute respiratory failure with an esophageal pressure monitor in place, greater changes in transpulmonary pressure within the first two hours of NIV were significantly associated with NIV failure and the need for intubation as well as the progression of infiltrates on a roentgenogram [22]. Although hardly definitive for P-SILI as the causative mechanism, such data suggest damaging strain due to excessive local stretch as a plausible explanation for their findings. Similarly, as applied to COVID-19 pneumonia, the increased respiratory drive of hypoxemic respiratory failure may theoretically promote damaging transpulmonary pressures and pulmonary vascular engorgement. When repeated for hours to days, these may contribute to the progressive lung injury that causes approximately one third of these patients who are hospitalized to develop ARDS. P-SILI might also delay the recovery of the ventilated patient; in this context, it is noteworthy that a prolonged duration of ventilator support often occurs with COVID-19 ARDS. Uncertainties that come with invoking P-SILI as the cause of the unusually rapid deterioration have been expressed including a lack of strong clinical evidence [23]. While clinicians amid a busy ICU service cannot be expected to insert esophageal manometers to closely monitor transpulmonary pressures in patients with NIV, it certainly is reasonable to note a patient’s ventilating frequency, depth of tidal breathing, physical signs of respiratory effort, and the pace with which these observations progress. We advocate for maintaining a high level of concern for patients with increasing respiratory drive on NIV, recognizing that vigorous spontaneous respirations themselves may be pathologic rather than homeostatic and may portend the need for urgent intervention [16].

## 5. Timing of Intubation in COVID-19 Pneumonia

The topic of timing of intubation in COVID-19 has been hotly debated. A definitive answer for the question as to when to intubate someone with severe COVID-19 respiratory illness remains elusive. Data relevant to this question come from retrospective observational studies or prospective cohort studies; a randomized clinical trial would be very difficult to conduct, given the innumerable factors that go into the decision to initiate invasive mechanical ventilation. Furthermore, published studies set different definitions for “early” vs. “late” intubation, which makes meta-analysis of the data challenging. Nevertheless, looking at the evidence we do have, there are mixed results. There have been few large studies which support early intubation to decrease mortality for patients with COVID-19 respiratory failure [24,25,26]. The largest of these, conducted by Manrique and colleagues [24], compared patients who underwent very early intubation (defined as intubation prior to ICU admission) to early intubation (patients who were intubated within the first 24 h of ICU admission) and to late intubation (occurring after 24 h of ICU admission). They found that those patients who had very early intubation and those patients who had late intubation were exposed to higher mortality risks than those who underwent early intubation. Patients in the ‘very early’ group were older and had higher APACHEII and SOFA scores compared to the other groups, while patients in the ‘early’ group were younger and had higher PaO_2_/FiO_2_ ratios than the patients in the ‘late’ group. It seems, therefore, that mortality differences may be partially explained by the severity of illness [24]. The STOP-COVID investigators also found a mortality risk reduction for those patients who were intubated on ICU days 1–2 compared to ICU days 3–7, but that investigation excluded those patients who were severely ill upon admission (PaO_2_/FiO_2_ < 100, pH < 7.0, Lactate > 10 mmol/L, three vasopressors or inotropes, or cardiac arrest) [26]. One might consider this group of patients to have been in the very early intubation group of the Manrique study [24] that had poorer outcomes than their later counterparts. Taken together, the evidence points to an optimal period for intubation benefitting those patients whose respiratory insufficiency continues to go unresolved or show a negative trend [27]. 

Other investigators have used alternative criteria to define early vs. late intubation, which might be more generalizable than ‘days post-ICU admission’. For example, Camous et al. analyzed mortality rates among three patient groups: those intubated before or on day one of initiating steroids; on days one to seven; and after day seven (defined as very late intubation) [28]. Those who were intubated more than seven days after the initiation of steroids, although a small portion of the total population, had increased odds of mortality compared to those intubated earlier [28]. Gonzalez et al. defined early vs. late intubation in terms of the timing after initiation of respiratory support [29]. Those who were intubated more than 48 h after first respiratory support (the delayed intubation group) had increased in-hospital mortality compared to those intubated earlier [29]. Interestingly, they also analyzed pulmonary sequelae in survivors in both groups and found that those who underwent delayed intubation had greater decreases in DLCO and worse imaging severity scores than those who experienced early intubation, suggesting that the timing of intubation may have lasting impacts on pulmonary function [29]. 

It is important to point out that these are observational and retrospective studies, and the possibility of a confounding factor amongst the early or late intubation group cannot be excluded. Indeed, there is an equal number of reports that have suggested no difference in mortality incidence between early and late intubation for severe COVID-19 respiratory illness [30,31]. In the largest of those, involving nearly 9000 patients, Papoutsi et al. conducted a systematic review and meta-analysis of cohort studies comparing early vs. late intubation strategies [31]. Early intubation was defined as occurring within 24 h from ICU admission, whereas late intubation comprised all patients intubated after 24 h of admission to the ICU. The authors found no difference in all-cause mortality or the duration of mechanical ventilation between the groups. Limitations of the study include significant heterogeneity among individual component studies and a lack of data concerning the clinical severity at the time of intubation [31]. While the data remain inconclusive, there may have been a collective signal for earlier intubation being protective in these patients.

In summary, the best timing for intubation in patients with severe COVID-19 pneumonia remains uncertain. The ROX index can be calculated quickly at the bedside and can suggest which patients currently receiving support via high-flow nasal cannula will later require invasive mechanical ventilation [32]. It might be useful to clinicians in triaging patients with impending respiratory failure, especially when resources such as invasive mechanical ventilation may be scarce. However, the authors caution against a prolonged trial of NIV in any instance if the patient trends towards more severe illness. In the end, the clinician must weigh the risks and benefits of invasive mechanical ventilation for the patient in front of them—a familiar application of the art of medicine that well pre-dates the onset of the pandemic. 

## 6. Awake Prone Position

During the initial period of the COVID-19 pandemic when hospitals and intensive care units were overwhelmed and advanced life support resources were limited, clinicians rightfully explored therapies which might help avoid the need for these interventions in their patients. In addition to careful monitoring on HFNC or NIV, trials of awake (or non-intubated) prone positioning were investigated. This practice had been explored prior to the onset of the COVID-19 pandemic in patients with other causes of ARDS. Small case series suggested that awake prone position was well tolerated, improved the PaO_2_/FiO_2_ ratio, and might avoid the need for intubation [33,34]. The huge volume of patients with ARDS during the COVID-19 pandemic allowed for the evaluation of the awake prone position in larger groups of patients. In a systematic review and meta-analysis of randomized controlled trials of awake prone positioning in patients with COVID-19 ARDS, a period of awake prone position the decreased need for intubation (RR 0.84; 95% CI 0.92–0.97), enabled the provision of necessary advanced respiratory support (HFNC or NIV), and/or was managed in an ICU setting [35]. Those patients who were not in an ICU setting or were receiving conventional oxygen support did not have similar reductions in their risk for intubation, perhaps related to less frequent monitoring [35]. 

Similar to the prone position in intubated patients, the mechanism of benefit in the awake prone position in patients with ARDS is likely improved ventilation perfusion matching. In prospective physiologic study of adults with COVID-19 pneumonia receiving conventional or high-flow oxygen, Liu and colleagues used electrical impedance tomography to assess the effect of the prone position on V/Q matching [36]. They found that, in spontaneously breathing supine patients with COVID-19 pneumonia, impaired V/Q matching was due, in greater part, to dead space than to shunting [36]. This finding is in keeping with the aforementioned mechanism of hypoxemia in these patients due to the disruption of pulmonary vascular regulation. The prone position improved V/Q matching through its tendency to decrease dead space. These benefits were limited to the time the patient was in the prone position and were lost when the patient resumed the supine position [36]. 

Such data indicate that the awake prone position improves V/Q matching while the patient maintains the prone position and suggest that it may help avoid the need for intubation in patients with COVID-19 pneumonia by allowing time for progress toward recovery and/or by limiting the need for high inspired concentrations of oxygen. 

## 7. Ventilator Management in COVID-19 ARDS

The overarching goal of mechanical ventilation for acute respiratory failure is to support the patient while allowing the lungs time to heal and to avoid further lung injury. The current pillars of conventional ventilator management in ARDS include limiting tidal volumes to 4–8 mL/kg predicted body weight (PBW) and inspiratory plateau pressures to less than 30 cmH_2_O, targeting relatively higher, as opposed to lower, expiratory pressures to maintain open lung units and prevent atelectrauma, and the use of prone positioning for patients experiencing refractory hypoxemia [37]. The principles that motivate these pillars of management remain uncontested, but their numerical guidelines may require adaptation to various phenotypes that can occur within the syndrome of ARDS. It has been reported that, on average, patients with COVID-19 ARDS have similar compliance to non-COVID-19 ARDS [38]; however, as already noted, there is a time-dependent evolution of the underlying pathology as well as responsiveness to ventilatory interventions [39]. It may be that some patients with COVID-19 ARDS do not benefit from and may adversely respond to traditional ARDS ventilator management, though additional studies are still needed to support this possibility. Nonetheless, it seems prudent that ventilator management in COVID-19 ARDS should take into account the nuances of atypical respiratory physiology and mechanics at the time of observation. Several pressing questions that we discuss subsequently arise during the ventilator management of COVID-19 ARDS. 

### 7.1. Tidal Volume 

What is the most appropriate tidal volume for patients with COVID-19 ARDS? Since the publication of the ARMA trial, starting tidal volumes for patients with ARDS have ranged from 4 to 8 mL/kg PBW, with 6 cc/kg PBW being a common target. It has been reported that, even in patients without ARDS, tidal volumes between 4 and 6 cc/kg PBW are associated with a lower risk of developing acute lung injury and lower mortality [40]. Setting *initial* tidal volumes around 6 cc/kg PBW in COVID-19 ARDS patients is reasonable as well. However, many patients with COVID-19 ARDS initially present with preserved compliance and an unusually low mechanical burden of pulmonary infiltrate and edema. These patients, classified as Type L by Gattinoni and colleagues, may be better served by using slightly higher tidal volumes in the 7–9 cc/kg PBW range [16]. Signs that a patient may benefit from these more liberal tidal volumes include well preserved respiratory system compliance, worsening respiratory acidosis, and spontaneous breathing efforts made above the ventilator-set targeted value at lower VT settings. Indeed, even in the landmark trial of 6 vs. 12 cc/kg PBW, tidal volumes assigned to the low VT cohort could be increased by caregivers to improve ventilator synchrony [19]. Whichever tidal volume strategy is chosen, we advocate for monitoring respiratory system compliance and driving pressures during passive breathing and for vigilance for worsening respiratory mechanics or ventilation ratios, at which time imposing a more conservative range of tidal volumes and/or higher PaCO_2_ may be appropriate to avoid injurious end-tidal stretching of the shrinking ‘baby lung’ [41].

### 7.2. Positive End Expiratory Pressure 

**Figure 3:**
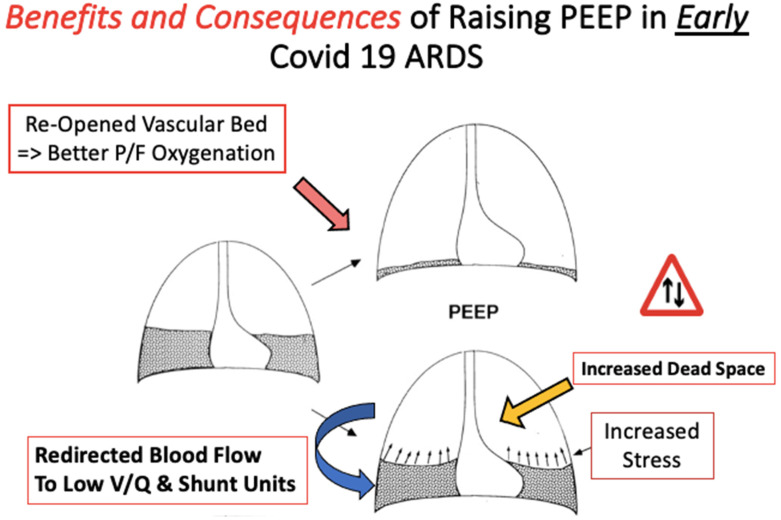
Benefits and consequences of raising PEEP in early COVID-19 ARDS. The goal of raising the PEEP is to open collapsed lung units (dark grey) that may participate in gas exchange and improve V/Q matching (depicted by the upwards arrow to the lungs with reduced consolidation). However, raising the PEEP may instead redirect blood flow to collapsed lung units and increase stress on the baby lung without the desired benefit of improved V/Q matching (depicted by the blue arrow). As the disease progresses, responses to the PEEP may change.

What is the most appropriate PEEP setting for patients with COVID-19 ARDS? In “typical” ARDS, in which alveolar edema, atelectasis, and low respiratory system compliance predominate, the purpose of applied end expiratory pressure is to increase the number of functional alveoli participating in gas exchange, to avoid tissue injury from cyclic tidal opening and re-closure, and to improve respiratory system compliance by distributing energy across a greater number of lung units. With this objective and atelectasis reversal in mind, guidelines for typical ARDS recommend targeting relatively higher, as compared to lower, PEEP in patients with ARDS [37]. The use of “PEEP Tables” to set FiO_2_ and PEEP is common practice and may specify PEEP values exceeding 14 cmH_2_O when the high-PEEP strategy is employed [42]. However, from the onset of the pandemic, it was recognized that many patients with COVID-19 ARDS may not respond to increases in PEEP in the fashion expected for traditionally encountered ARDS (Figure 3). Barthelemy et al. published a retrospective study of 30 patients during the first wave of the pandemic in which they assessed responses in PaO_2_/FiO_2_ ratios, respiratory system compliance (Crs), cardiac output, and oxygen delivery to incremental increases in PEEP [43]. Those investigators found that, while PaO_2_/FiO_2_ ratios increased by an average of 10 mmHg for each 1 cmH_2_O increase in PEEP, Crs and cardiac output both declined in response to rising PEEP. The overall effect was decreased systemic oxygen delivery. The authors concluded that higher recommended levels of PEEP could be unbeneficial to some patients with COVID-19, an observation corroborated by the work of others [43]. Chiumello and colleagues documented the effects of “low” vs. “moderate” PEEP (5 vs. 15 cmH_2_O respectively) in 61 patients with early-phase COVID-19 ARDS and relatively preserved compliance (44 mL/cmH_2_O). While PaO_2_/FiO_2_ predictably improved from 5 to 15 cmH_2_O PEEP, the calculated lung stress and mechanical power of ventilation also improved [44]. The study concluded that the heterogeneity of respiratory mechanics amongst patients with COVID-19 ARDS and the unique underlying pathophysiology driving its disordered hypoxemia make it difficult to predict key responses to PEEP titration. In this study, the “empiric PEEP”, as set by the PEEP/FiO_2_ table [42], was 18 cmH_2_O. Significant increases in driving pressure, lung stress, and mechanical power with empiric PEEP, as compared to 15 cmH_2_O, argued against using traditional PEEP tables for determining optimum PEEP levels in COVID-19 ARDS. Ultimately, the authors supported tailoring PEEP to the individual patient, recognizing that the most prudent level of PEEP is likely to change over the course of a patient’s respiratory illness [44]. When doing so, titrating PEEP using respiratory system static compliance seems a reasonable guide [45]. Increases in PEEP that improve respiratory system compliance at a constant tidal volume represent net lung recruitment as opposed to the overdistention often encountered at higher levels of support. The ventilatory ratio offers another parameter that can be easily tracked and used to support decisions to increase or reduce PEEP [46]. If increases in PEEP recruit additional functional lung units without being outweighed by overdistention, physiologic dead space decreases and the ventilatory ratio improves. 

### 7.3. Prone Position 

**Figure 4:**
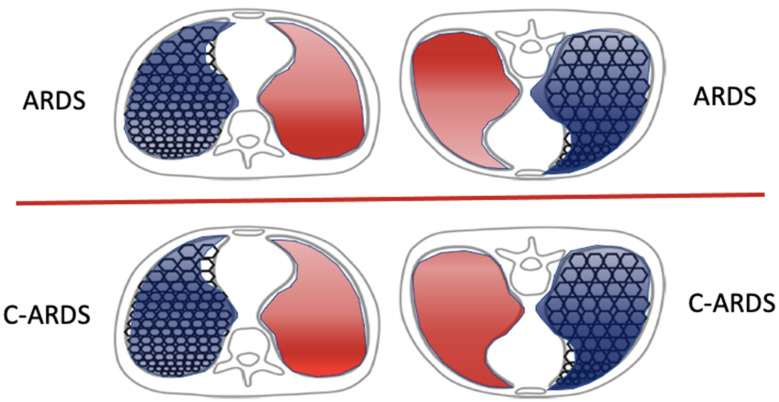
Effects of prone positioning on the distributions of ventilation (blue shading) and perfusion (red shading), contrasting COVID-19 ARDS versus traditional ARDS. Ventilating patterns are affected similarly by prone positioning in both categories of ARDS. However, while regional perfusion remains relatively unaltered by prone positioning in traditional ARDS, gravitational forces combined with impaired homeostatic Vaso regulation alter regional perfusion in C-ARDS.

Does prone positioning benefit patients with COVID-19 ARDS? Since it was first reported as clinically useful in the mid-1970s, prone positioning of ventilated patients with ARDS has become a routine practice for patients with moderate to severe ARDS. Its widespread adoption for this subset of traditional ARDS followed the demonstration of its benefit to mortality risk [47]. The anterior ventral portions of the chest wall are innately more flexible than the posterior dorsal ones. Prone positioning, which braces the anterior chest and helps even the regional distribution of transpulmonary pressure [48], has several beneficial physiologic effects that often result in improved oxygenation and a reduced VILI hazard. Firstly, it recruits the previously collapsed and richly perfused dependent dorsal lung units and partially relieves the cardiac compression of those regions experienced when supine [49]. The recruitment of dorsal lung units generally exceeds the de-recruitment of ventral lung units, promoting a more functional, open lung. Furthermore, perfusion is not adversely affected by the associated gravitational changes; dorsal lung units continue to receive a higher proportion of total blood flow compared to ventral units when prone. This recruitment of dorsal lung units with preserved or even increased blood flow due to the relief of local hypoxic vasoconstriction improves ventilation/perfusion matching and oxygenating efficiency [50]. The use of lower PEEP and/or FiO_2_ may follow. The reduction in mortality risk may also stem from the more homogenous distribution of ventilation and decreased local stress and strain applied to already injured lungs [50]. With recognition of the unexpectedly adverse consequences of PEEP and of better tolerance to non-invasive measures for ventilation, the pandemic dramatically increased the percentage of COVID-19 ARDS patients managed in the prone position. It should be recognized that the prone position is the natural one in the great majority of mammals for whom sustaining any supine orientation over hours is highly unusual. In fact, prone and semi-prone postures are routinely adopted by many healthy persons during sleep [51]. In one early report that included over one thousand patients with COVID-19 from 24 different Italian ICU’s, 61% were treated using prone positioning at some point during their ICU stay [52]. The authors found that prone positioning of those patients yielded a significant (>20 mmHg) increase in the PaO_2_/FiO_2_ ratio but did not change respiratory system compliance or the ventilatory ratio [52]. Using electrical impedance tomography, Fossali et al. [53] demonstrated that the underlying physiologic benefits of proning in COVID-19 are, in some ways, similar to those of traditional ARDS: namely, increased lung recruitment and improved ventilation–perfusion matching. Yet, detailed investigations of COVID-19 ARDS have called attention to the unusually strong influence of gravity on the responses of a patient’s dysregulated pulmonary vasculature [16] (Figure 4). Indeed, the expected oxygenation benefit from prone positioning may not be realized in many. In COVID-19 ARDS, the timing of proning appears to be of major clinical importance for oxygenation as well as for lung compliance. One international cooperative study of 376 patients with COVID-19 ARDS and non-COVID-19 ARDS (220 and 156, respectively) found, in both cohorts, that those who underwent prone positioning within 24 h of intubation experienced greater improvements in oxygenation and greater benefits to their mortality risk, as compared to patients who were proned later in their intubation course [54]. In later stages of ARDS, the prone position is less likely to benefit patients, as the lung tissue becomes increasingly organized toward fibrosis, the gas volume diminishes, and the potential for recruitment fades [55]. 

### 7.4. Detecting End-Tidal Hyperinflation

**Figure 5:**
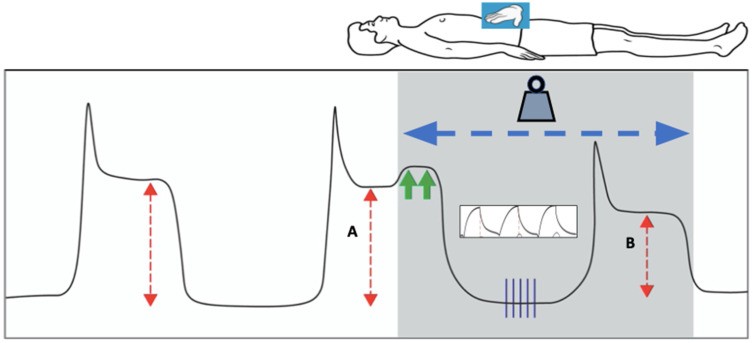
Paradoxical effects of loading via horizontal positioning or external abdominal pressure on respiratory system compliance, as exemplified by plateau pressure with an unchanging tidal volume and PEEP. Constant manual pressure is applied to the abdomen with enough force to raise the plateau pressure by two centimeters of water (green arrows). Upon resumption of tidal breathing, three to five breath cycles (vertical blue lines) are delivered under loaded conditions (shaded) with the same constant force applied to the abdomen, and a new plateau pressure is measured. If it is observed to be lower than the original plateau pressure while under the load, this is consistent with mechanical paradox. The red arrows depict the difference in plateau pressure between pre-loaded and loaded conditions (A: P_plat_, B: P_load_). Note that loading can also be applied without the need for compression maneuvers via the simple transition from upright to horizontal positioning of the thorax.

How can net overdistention be detected at the bedside in patients with COVID-19 ARDS? It is widely understood that the pressures, volumes, and flows monitored at the airway opening apply their energy to a heterogeneous mechanical environment. Not only the lung but also the chest wall expands, and regional compliance varies with regional anatomy, position, and pathology. Determining whether a current ventilator prescription is causing widespread lung hyperinflation with hazardous parenchymal stress and strain has been a long-sought goal in ARDS management. PEEP titration protocols [56] and assessment of the stress index of the pressure volume curve during passive-volume-controlled, square-wave (constant flow) ventilation [57] are two available ways in which a clinician might determine net end-tidal hyperinflation; but, without specialized equipment, these methods require interventional measures and may be time-consuming. As discussed earlier, the chest wall forcefully resists compression below its natural resting volume, and transpulmonary lung pressures decline with the accompanying rise in pleural pressure. As a consequence, respiratory system compliance is not expected to improve during external compression. With this principle in mind, assessing respiratory system compliance in response to brief but sustained chest wall compression (lasting < 15 s) has gained attention as another simple method for detecting net end-tidal hyperinflation during the COVID-19 pandemic [58]. Manual compression sustained for several breathing cycles produces effects which are immediate, quickly reversed, and universally available (Figure 5). Actually, a variant of this practice pre-dates the pandemic: two case reports exist in which investigators laid weights on the anterior chest in supine trauma patients with ARDS who could not be placed in the prone position [59]. In each report, the authors found improved, rather than the expected reduction in, respiratory system compliance and postulated that chest wall weighting led to increased dorsal lung ventilation and improved ventilation/perfusion matching. More recently, multiple reports during the COVID-19 pandemic have noted ‘paradoxically increased’ respiratory system compliance with either chest wall or abdominal compression of patients with late-stage, low-compliance ARDS due to COVID-19 [60,61,62,63]. The aerated capacity of these very small, late-stage ‘baby lungs’ as well as the potential for recruitment by higher transpulmonary pressures are greatly reduced. Indeed, deterioration in gas exchange or maneuver-associated decruitment has not been reported in patients with paradox. Of note, while prone positioning itself routinely stiffens the chest wall and routine ARDS does not usually alter global compliance, further external compression of the chest wall in severe COVID-19 ARDS may also confer mechanical benefits [61]. 

The suspected physiologic mechanism by which external chest wall compression improves tidal compliance of the respiratory system in these patients is by decreasing the total aerated volume of the ARDS lung. Doing so allows the remaining open lung units to operate on a more linear and less distended portion of their pressure–volume curves. The driving pressure then sinks below the upper inflection zone of reduced tidal compliance and puts the patient at greater risk of overstretching [64]. Similar improvements in respiratory system compliance are found in those patients who demonstrate mechanical paradox when decreasing the PEEP or tidal volume [65]. Along similar lines, by altering the chest wall compliance, transpulmonary pressure, and lung volume, body positioning may influence respiratory system compliance in unexpected ways. Selickman and colleagues demonstrated that, in severe, late-stage COVID-19 ARDS, patients who demonstrated mechanical paradox, bed inclination towards a more upright position decreased rather than improved respiratory system compliance—often markedly [66]. That unexpected effect of routine “head up” clinical practice could be reversed via abdominal compression. It follows that more horizontal body positioning or abdominal binding when upright might be considered as a ‘lung-protective’ intervention for these patients. Future studies are needed to assess the durability of any such benefits as well as the impact of chest wall compression on respiratory system compliance in less severe ARDS as well as in settings of respiratory insufficiency occurring in non-injured lungs. These immediately reversible interventions may hold diagnostic benefits by providing simple maneuvers with which the bedside clinician can quickly detect excessive stress on the lungs due to mechanical ventilation (Figure 5). It has been reported that compressive benefits for tidal mechanics may be sustained in the supine orientation for hours or days without apparent deterioration in mechanics or gas exchange. Perhaps this is not terribly surprising, as the compression of prone positioning is well tolerated over similar periods. Yet, sustained tolerance may depend on the reduced tendency to de-recruit in small, late-stage ‘baby lungs’ with mechanical paradox. In summary, transient compression and repositioning maneuvers are quickly applied, well-tolerated, immediately reversed, and universally available. These hold unquestioned diagnostic value for implementing a truly “lung-protective” strategy. Thus far, however, sustained external chest wall restriction has not been convincingly shown to offer therapeutic outcome benefits to those with mechanical paradox [67]. 

## 8. Extracorporeal Membrane Oxygenation for COVID-19 ARDS 

Does ECMO improve the chance for lung healing in severe COVID-19 ARDS? All measures that reduce metabolic or volitional ventilatory demand have the potential to facilitate lung protection by lowering the damaging energy applied to acutely injured lungs. Improved oxygenation also reduces the need for elevated concentrations of inspired oxygen, high mean airway pressures, and tidal stresses that impose VILI risks. Veno-venous extracorporeal membrane oxygenation (VV-ECMO) offers these advantages and has been successfully used for patients with severe ARDS well before the COVID-19 pandemic. 

The criteria for the initiation of ECMO for patients with severe ARDS come from the EOLIA trial [68]. They include a PaO_2_/FiO_2_ ratio less than 50 mmHg for more than three hours, a PaO_2_/FiO_2_ ratio less than 80 mmHg for more than six hours, or an arterial pH < 7.25 with a PaCO_2_ of 60 mmHg or greater for more than six hours [68]. Although the primary outcome of mortality at 60 days between ECMO and control groups was not statistically significant, approximately one third of the control group crossed over to the ECMO group due to refractory hypoxemia, which likely affected the results [68]. Importantly, other strategies for the management of severe ARDS must have been attempted prior to considering a patient for ECMO, as outlined by the Extracorporeal Life Support Organization (ELSO) guidelines. These include standard lung-protective ventilation; prone positioning; and consideration of a high-PEEP trial, neuromuscular blockage, or inhaled pulmonary vasodilators. Furthermore, participants had received mechanical ventilation for less than 7 days at the time of enrollment. Potential contraindications to receiving ECMO include advanced age, BMI > 45, intubation for >10 days prior to enrollment, chronic respiratory failure, heart failure requiring veno-arterial (VA) ECMO, severe acute multiple organ failure with anticipated death despite ECMO, severe acute neurologic injury with poor prognosis for recovery, patient refusal of blood products, inability to receive anticoagulation, and expected limitations to vascular access for cannulation [68,69]. The current guidelines for initiating ECMO for COVID-19 are in keeping with these pre-existing ECMO guidelines, with a few contingency plans based on capacity during a pandemic [69]. 

Spurred by the need to improve gas exchange efficiency in lungs resistant to conventional and less aggressive measures, the use of VV-ECMO rapidly expanded during the COVID-19 pandemic. Valuable experience with extracorporeal support was gained as a beneficial consequence. As was previously shown in the EOLIA trial, COVID-19 patients treated with ECMO required lower fractions of inspired oxygen and had lower respiratory rates, tidal volumes, plateau and driving pressures, and mechanical power [68,70,71]. In theory, this ‘lung rest’ decreases the risk of ventilator-induced lung injury, allowing the lungs time to heal from the immunologic insult. 

The earliest reports of outcomes for patients with COVID-19 ARDS who received ECMO were quite grim when compared to conservative ARDS management [72]. However, more recent and larger systematic reviews have shown that ECMO outcomes for patients with COVID-19 ARDS are similar to the non-COVID-19 ARDS cohort of the EOLIA trial. A meta-analysis of 22 observational studies including over 1800 patients during the first wave of the pandemic found that in-hospital mortality for patients receiving ECMO for COVID-19 respiratory failure was 37.1% [73]. A larger analysis including 52 studies and over 18,000 patients enrolled from 2020 to 2021 found a pooled mortality rate of 49% for patients receiving ECMO for COVID-19 ARDS [74]. Notably, this mortality spanned the longest reported time of follow up, rather than the in-hospital or ICU mortality reported in other studies. Increasing age, time of enrollment (especially the first half of 2021), and the proportion of patients receiving corticosteroids were all associated with increased mortality risks. Herrmann et al. evaluated key characteristics associated with survival for COVID-19 ARDS treated with ECMO. They included 673 patients from 26 different centers in Germany over the first two waves of the pandemic. Overall, the ICU survival for patients treated with ECMO was 31.4%. Importantly, survival was higher for patients meeting modified EOLIA criteria (age 70 or younger, BMI 45 or lower, mechanical ventilation for less than 8 days at the time of ECMO initiation, absence of malignancy, and no medical history of myocardial infarction, congestive heart failure, chronic pulmonary disease, or moderate to severe liver disease), for younger patients, for patients who received ECMO less than 5 days after intubation, and for patients who were treated at high-volume ECMO centers [75]. Overall, ECMO appears beneficial for well-selected patients with severe ARDS due to COVID-19, when appropriately applied in a timely manner. Initiating ECMO early in the disease course to minimize lung injury from damaging mechanical power offers an attractive, even if unproven, rationale. 

## 9. Conclusions

In conclusion, COVID-19 ARDS has challenged and expanded our understanding of acute respiratory distress syndrome and best management practices. Outlined below are important lessons we have learned during the pandemic to improve the care of patients with COVID-19-related respiratory failure. 

### 9.1. Oxygenation-Based Criteria for ARDS May Misdirect Ventilation Strategy

COVID-19 represents a distinct etiology of acute lung injury whose hallmark feature is conspicuous injury to the pulmonary vascular compartment, leading to vasomotor dysregulation, endothelial injury, and thrombosis. Clinically, this can manifest initially as severely impaired gas exchange with relatively little parenchymal involvement in radiographically and relatively preserved respiratory system compliance.

### 9.2. Mechanical Properties May Change Dramatically over Time

The timing of intubation in COVID-19 respiratory disease must weigh the risks of ventilator-induced lung injury and other side effects of mechanical ventilation against the risks of prolonged non-invasive respiratory support and likely development of self-inflicted lung injury (P-SILI). A brief trial of non-invasive ventilation may avoid intubation when dyspnea and vigorous breathing efforts convincingly subside. But, caution must be exercised during a prolonged trial, especially if the perceived work of breathing is excessive or is not improving with non-invasive ventilatory support.

### 9.3. Personalize Ventilator Settings

These must be adjusted to the needs of the individual patient. In our experience, patients earlier in their disease course of COVID-19 may better tolerate higher tidal volumes and lower PEEP levels than recommended by standard ARDS protocols and published tables. Daily reassessment of respiratory system compliance is especially important as, over time, unresolving COVID pneumonia transitions into more traditional ARDS physiology followed by a progressively shrinking baby lung and less compliant respiratory system. 

### 9.4. Challenge Traditional Assumptions Regarding Lung Protection

Atypical respiratory mechanics were frequently encountered in severe, late-stage COVID-19 pneumonia. During the pandemic, novel and readily implemented methods for detecting net end-tidal overdistention were described that involve body position changes or temporarily applying external pressure to the chest wall or abdomen. With tidal volume unchanged, ‘paradoxically’ improved respiratory system compliance during chest wall loading implies that the remaining ‘baby lung’ lung units should operate on a less overstretched portion of their pressure–volume curves. 

### 9.5. Prone Positioning May Forestall or Negate the Need for Intubation

The prone position may yield benefits during spontaneous breathing and often (but inconsistently) improved oxygenation in patients with COVID-19 ARDS when implemented earlier in the course of mechanical ventilation. The mechanism of proning for gas exchange in that setting had atypical features, whereas it predictably improved the uniformity of transpulmonary pressure distribution. As lung injury progresses, the benefits of proning for gas exchange diminish. 

### 9.6. The COVID-19 Pandemic Expanded Our Experience with Venovenous ECMO for ARDS Treatment

Use of this demanding methodology has been more safely and widely deployed. Extracorporeal gas exchange proved useful for severe ARDS due to COVID-19 when provided to appropriately selected patients in a timely manner. Its use earlier in the disease course allows for lung rest and the application less damaging ventilator power to already injured lungs.
